# Environmental impacts on single-cell variation within a ubiquitous diatom: The role of growth rate

**DOI:** 10.1371/journal.pone.0251213

**Published:** 2021-05-07

**Authors:** Elisabeth Groß, Maarten Boersma, Cédric Léo Meunier

**Affiliations:** 1 Alfred-Wegener-Institut Helmholtz-Zentrum für Polar- und Meeresforschung, Biologische Anstalt Helgoland, Helgoland, Germany; 2 University of Bremen, FB2, Bremen, Germany; CSIR-National Institute of Oceanography, INDIA

## Abstract

Morphological and physiological characteristics of phytoplankton cells are highly sensitive to changes in environmental conditions and, in turn, influence the dynamics of phytoplankton populations and communities. To cope with environmental change, trait variability and phenotypic plasticity may play an important role. Since global change comprises simultaneous changes in abiotic parameters, we assessed the impact of multiple drivers on functional traits of the diatom *Thalassiosira (Conticribra) weissflogii* by manipulating concurrently temperature, pCO_2_, and dissolved nitrogen:phosphorus (N:P) ratio. We tested three scenarios: ambient (ambient temperature and atmospheric pCO_2_; 16 N:P ratio), moderate future scenario (+1.5°C and 800 ppm CO_2_; 25 N:P ratio), and more severe future scenario (+3°C and 1000 ppm CO_2_; 25 N:P ratio). We applied flow cytometry to measure on single-cell levels to investigate trait variability and phenotypic plasticity within one strain of diatoms. Growth rates differed significantly between the treatments and were strongly correlated with cell size and cellular chlorophyll *a* content. We observed a negative correlation of growth rate with chlorophyll *a* variability among single strain populations and a negative correlation with the phenotypic plasticity of cell size, i.e. when growth rates were higher, the cell size cell-to-cell variability within cultures was lower. Additionally, the phenotypic plasticity in cell size was lower under the global change scenarios. Overall, our study shows that multiple traits are interlinked and driven by growth rate and that this interconnection may partly be shaped by environmental factors.

## Introduction

Phytoplankton are responsible for about 50% of Earth’s primary production [1], with diatoms accounting for about half of this [2,3]. As primary producers, phytoplankton form the basis of most marine food webs. In addition, diatoms and phytoplankton in general can serve as indicators of ecosystem change since they are particularly sensitive to shifts in environmental conditions. This is also important in the context of regional and global change, since human activities and associated increases in greenhouse gas emissions have led to simultaneous changes in a number of aquatic abiotic parameters. Consequently, phytoplankton are exposed to the simultaneous effects of multiple anthropogenic stressors, such as increasing temperature and pCO_2_, as well as shifts in dissolved nutrient concentrations and stoichiometry.

Phytoplankton can respond to changes in environmental conditions at different levels. At the first level, the phenotypic plasticity of individual phytoplankton cells largely determines whether the cells can subsist under changing abiotic conditions. How well a single strain copes with changing environmental conditions depends on the tolerance range of individual cells [4,5]. At the second level, the plasticity of different strains within a population is the critical factor. High plasticity of individual strains has the potential to buffer negative effects of environmental change, while low plasticity of these strains can lead to shifts in population structure [6]. At this level, the sum of the tolerance ranges of different strains within a population determines whether the population can cope with environmental change. The third level of phytoplankton response results in a change in community composition when altered abiotic conditions lead to species selection. Despite the importance of all three levels of response (within strains, between strains, between species), many studies have focused on the response of community [7–9], populations [10,11], and strains [6,12,13], while much less attention has been paid to the phenotypic plasticity of individual cells within a strain.

One of the most important characteristics or traits of individual phytoplankton cells that determine their ecological success is cell size [reviewed by 7], as it is directly correlated with many other traits and is influenced by a range of environmental factors. Smaller cells have lower sinking rates [14], better nutrient uptake due to a higher surface-to-volume ratio, and a smaller diffusive boundary layer [15,16]. On the other hand, smaller cells are often more susceptible to grazing [17]. Thus, cellular characteristics not only affect the ecological role of plankton but are also shaped in part by environmental conditions. Changing temperature and pCO_2_ regimes could, therefore, have an impact on cellular traits, with consequences for trophic and system processes. So far, most studies of phytoplankton traits and their response to environmental drivers have been conducted using bulk measurements. For example, Hillebrand et al. [18] observed that phytoplankton exhibit large variability in cellular nutrient content at low growth rates and that cellular nitrogen to phosphorus (N:P) ratios converge with increasing growth rate. Hence, faster growth causes cultures to become more similar and their variability to decrease. This phenomenon was first observed by Goldman in 1986 [19]. However, the study by Hillebrand et al. compared different populations and different species, and although the authors conclude that this should be the case, it remains unclear whether the above-mentioned patterns also apply within individual strains. Given the importance of phenotypic plasticity in coping with altered environmental conditions, a proper evaluation of the response of phytoplankton to global change cannot be made without studying their phenotypic plasticity and cell-to-cell variability within individual strains. Flow cytometry, which measures traits of individual cells, is a well-established method to investigate the variability of cellular characteristics such as cell size, pigments, and biochemical components [20–22].

Since current CO_2_ concentrations are not saturating for Rubisco, the enzyme that catalyzes primary fixation of inorganic carbon [23], higher pCO_2_ resulting from anthropogenic emissions can potentially favor photosynthesis and phytoplankton growth [24–26]. Furthermore, rising water temperatures caused by increased atmospheric CO_2_ can affect physiological processes, including growth, resource acquisition, and photosynthesis [27,28]. Additionally, human activities influence the concentrations and ratios of dissolved nutrients such as nitrogen (N) and phosphorus (P), which are essential for phytoplankton growth. Legal restrictions on nutrient inputs to the environment have resulted in increasing N:P ratios, which are predicted to rise even further in the future, as atmospheric N deposition is likely to continue to increase [29], and P reserves on Earth could be depleted in 50 to 200 years if current levels of use are maintained or increased [30,31]. However, as environmental conditions change, the elemental stoichiometry of phytoplankton species and their biochemical requirements will also be affected [25]. Given the influence of these global change drivers on phytoplankton growth rate and the strong correlation of growth rate with other traits such as cell size [25], it is crucial to investigate the phenotypic plasticity of multiple phytoplankton traits, and how the traits are correlated.

Considering that changes in pCO_2_, temperature, and nutrient concentrations do not happen separately, but rather simultaneously, we conducted a multi-driver experiment with realistic future conditions based on predictions from the Intergovernmental Panel on Climate Change (IPCC). We tested the RCP 6.0 and 8.5 scenarios by simulating a temperature increase of 1.5 and 3°C combined with increasing pCO_2_ up to 800 and 1000 ppm, respectively, and we combined these scenarios with an increase in dissolved N:P ratios from 16 to 25. Using a single-cell approach, we analyzed the response of a single strain of the diatom species *Thalassiosira weissflogii*, currently also named *Conticribra weissflogii*. Since we acquired the strain we worked with from an algal culture bank under the name *T*. *weissflogii*, we will retain the generic name *Thalassiosira* in this paper. *T*. *weissflogii* is mostly found in temperate brackish waters, but it has also been isolated all around the globe in areas ranging from the Long Island Sound, New York, USA, to Jakarta Harbor, Indonesia, to the German Bight (North Sea), as well as in the middle of the Pacific Ocean [32–34] and can, therefore, be considered as a cosmopolitan species. Furthermore, *T*. *weissflogii* is known to react in a number of ways to altered abiotic conditions [35,36]. Sugie and Yoshima [37] reported a decrease in cell size of *T*. *weissflogii* under increased pCO_2_ and Wu et al. [38] identified a slight increase of growth rate at higher pCO_2_. Nevertheless, the single-cell plasticity of multiple traits of *T*. *weissflogii* has never been investigated. Since measurements on a single-cell level require certain criteria regarding cell size and chain-formation, we used this well-studied diatom species to analyze the link between growth rate, cell size, and biochemical composition of cells growing under different future global change conditions. More specifically, we tested the hypothesis that not only between species and populations [18] but also within one single strain, cell-to-cell variability of phytoplankton decreases with increasing growth rate. Using flow cytometry, we are able to analyze both, the reaction of single strain populations to different environmental conditions by studying the variation between cultures, as well as the variability within one replicate at a distinct growth rate. The former, we describe in the following as among-strain population variability and the latter as cell-to-cell variability within a single strain population. Both concepts can be used to assess phenotypic plasticity which plays an important role when addressing the question of the effect of global change on phytoplankton.

## Materials and methods

### Experimental setup

A single strain stock culture of *T*. *weissflogii* (Grunow) Fryxell & Hasle (obtained from the Culture Collection of Algae at Göttingen University, SAG, strain number 122.79) was grown in semi-continuous dilute-batch culture. This stock culture was diluted by 95% every 4–5 days with f/2 medium [39] prepared with artificial seawater. The inoculation stock culture was maintained under a 14:10 day:night cycle at 14°C and irradiance of 95μmol photons m^-2^ s^-1^ (GHL Mitras Lightbar 2 Daylight, 6500K, dimmed to 80%). All bottles were gently homogenized manually several times a day, which prevented sedimentation of the cells.

In a multi-driver experiment, we tested six scenarios based on IPCC predictions for the end of the 21^st^ century. It is important to note that our main interest was not to investigate the effects of the different stressors individually, but rather to create the complete scenarios, since temperature is not going to change without CO_2_ concentrations and vice versa. These combinations were crossed with two different nutrient scenarios, as the direction of change in nutrient stoichiometry is less well established in the current literature. Since the strain used in our experiment was maintained at 14°C for many months, which is within the optimal growth temperature range of *T*. *weissflogii* [40], we used this temperature as a reference for the “ambient” scenario. Hence, temperature and pCO_2_ levels were chosen to represent ambient conditions (amb = 14°C and 450ppm CO_2_), a moderate global change scenario based on RCP 6.0 (rcp6.0 = 15.5°C and 800ppm CO_2_), and a more severe global change scenario based on RCP 8.5 (rcp8.5 = 17°C and 1000ppm CO_2_). Since dissolved phosphorus concentrations are expected to decrease relative to nitrogen concentrations in the future, resulting in increased N:P ratio [29], these three treatments were crossed with **N**:**P** = 16 (Redfield ratio) and **N**:p = 25 (increased N:P ratio). We used batch cultures, as we were also interested in the dynamics of cellular traits over different growth phases. This enabled us to study the impact of global change scenarios on the one hand and the effects of shifts from exponential growth to stationary phase and from nutrient-replete to nutrient-depleted conditions on the other.

The experiment was initiated by inoculating 1L glass bottles with the stock culture at a starting cell density of 1000 cells ml^-1^ in six biological replicates for each treatment, yielding a total of 6 times 6 = 36 bottles. Each culture can be seen as a single strain population. We prepared the culture medium with artificial seawater based on f/2 medium [39]. The N concentration was 880 μmol L^-1^ and the P concentrations were 54 and 35 μmol L^-1^ for the **N**:**P** 16 and **N**:p 25 treatments, respectively. To maintain constant temperatures, the experiment was conducted in temperature-controlled rooms at 14.0, 15.5, and 17.0°C. To reach the desired pCO_2_, we aerated the culture medium with a mixture of air stripped of CO_2_ by soda lime and pure CO_2_ for 24 h before inoculation. At early and late stationary phase, we took samples for total alkalinity. Due to the high volume required for this analysis, we used two of the six replicates for both analyses. For each alkalinity sample, 125ml from the culture were filtered with a 0.45μm nylon filter (Sartorius) and stored airtight in borosilicate bottles at 4°C. We used the software CO2calc v4.0.9 to calculate the initial pCO_2_ in our treatments based on the measured alkalinity and the corresponding pH (amb_i_ = 412 ppm, rcp6.0_i_ = 776 ppm, rcp8.5_i_ = 922 ppm) as well as at early stationary phase at day 5 (amb_5_ = 76 ppm, rcp6.0_5_ = 64 ppm, rcp8.5_5_ = 17 ppm) and the late stationary phase at day 10 (amb_10_ = 1 ppm, rcp6.0_10_ = 1 ppm, rcp8.5_10_ = 1 ppm). Macronutrients were measured at the same time points (S1 Table).

After inoculation, *T*. *weissflogii* was grown in batch cultures for 10 days under the same light conditions as the stock culture. We sampled two milliliters of each biological replicate daily one hour after artificial sunrise and analyzed them directly via flow cytometry to evaluate how different traits are linked during different growth phases. Immediately after each measurement, we fixed the samples with acidic Lugol’s iodine solution at a final concentration of 2%, since Lugol is less hazardous than aldehyde-based or more toxic fixatives and does not cause cellular damage due to intracellular freezing. These samples were stored in the dark for subsequent flow cytometric analyses of cellular lipid and protein content.

### Flow cytometry

Fresh samples were analyzed daily using a flow cytometer (BD Bioscience Accuri C6) to determine the cell density of each culture. We processed the samples by running the flow cytometer at a medium flow rate (35 μl min^-1^). Volumes of 102 and 68 μl were measured for samples taken during exponential growth and stationary phase, respectively. This procedure ensured that at least 200 diatom cells of each sample were counted. The cell density was used to calculate growth rates for each replicate by the following equation:

$$\mu =\text{ln}\left(N_{t}/N_{0}\right)∙\Delta t^{-1}$$

whereby *N*_*0*_ and *N*_*t*_ describe cell concentrations at t0 and t and *Δt* is 1 since we measured every day.

To analyze correlations between growth rate and other functional traits, we used flow cytometric measurements of the same samples to detect changes in the cell size of *T*. *weissflogii* based on the forward scatter (FSC) and the chlorophyll *a* cell content using the FL3 (670+ nm) and FL4 (675/25 nm) fluorescence detectors. The fluorescence detectors FL3 and FL4 differ in their selective transmission of the fluorescence signals. Whereas FL3 (670 nm) is a longpass optical filter, the FL4 (675/25 nm) is a bandpass filter. The main Chl a fluorescence band of diatoms is at 685/695 nm of the photosystem II, which means that both detectors cover the Chl a signal but FL3 is more distinct with a coverage between 650 and 700 nm whereas FL4 also includes the fluorescence signal of the photosystem I at 720/740 nm of the cells [41]. While the different detectors do not give absolute values of size and chlorophyll *a*, here, we were interested in the variation between and within treatments rather than absolute changes in parameters. Consequently, we measured cell characteristics with arbitrary units, which is a well-established way to deal with flow cytometric measurements, with forward scatter correlating with cell size and the 670 nm fluorescence signal correlating closely with the cellular chlorophyll *a* content [reviewed by 42]. Since the natural fluorescence of cells is sensitive to light and temperature, we took samples of 2 ml each from six cultures at a time, followed by immediate measurement and fixation. The side scatter (SSC) detects granularity of cells. Every cell is measured individually using flow cytometry. Therefore, with each measurement, not only the mean value of each parameter of a sample was recorded, but also the coefficient of variation (*CV* = (*SD*/*mean*) · 100) of the sample to determine the cell-to-cell variability. We used the flow cytometric results to analyze the variability among single strain populations as well as cell-to-cell variability within a single strain population. For the former, we used the mean values of each parameter from the different flow cytometry detectors and analyzed the variance of each trait among cultures, and for the latter, we used the coefficient of variation (CV) of cellular parameters.

### Staining

Samples from days 1, 3, 5, 6, and 10 were used for cellular lipid and protein analysis, representing exponential growth and early and late stationary phase. To analyze cellular lipid and protein contents, we used the stains Nile Red (Thermo Fisher Scientific) and Fluorescein-5-Maleimide (FITC, Thermo Fisher Scientific) [43,44], respectively. Nile Red can be used to detect both polar (membrane) lipids, stained in red, and non-polar lipids, which are present in lipid droplets and stained in yellow [45]. The Nile Red working solution was prepared each day by diluting an initial stock solution (10 mg ml^-1^) with acetone to a concentration of 2.5 μg ml^-1^. The FITC working solution for protein staining was prepared fresh each day from the stock solution by dissolving and diluting with ethanol to a concentration of 5 μg ml^-1^. For each of the stains, we used 225 μl of the Lugol-fixed samples. The color of the iodide was removed by adding 12.5 μl of sodium thiosulfate solution [46] directly before staining. Afterward, 25 μl of the working solution of either Nile Red or FITC was added to the sample. The incubation of the stains was performed in black Eppendorf tubes for 30 min, as both the optimal incubation time and stain quantity were determined in a preliminary experiment. Samples were measured by flow cytometry at a low flow rate (14 μl min^-1^) and a sample volume of 41μl was measured. Since the cell concentration was still quite low at day 1 and fewer cells were detected with staining than in fresh samples, the measured sample size ranged from 70 to 100. The staining signal of Nile Red was detected using FL2 (585/40 nm) and FL3 (670+ nm) fluorescence detectors for the non-polar and polar lipids. The signal from FITC was detected with the fluorescence detector FL1 (533/30 nm). A control was measured from each sample to correct the staining signals of Nile Red and FITC with the natural fluorescence signal of the diatom cells.

### Data analysis

The software R [47] was used for all statistical analyses. Differences between the treatments were tested using analysis of variance (two-way ANOVA) and Tukey’s (HSD) *post-hoc* comparisons, with IPCC scenario (ambient, 6.0, and 8.5) and nutrient scenario as independent factors. For all statistical comparisons, normality (Shapiro) and variance homogeneity (Barlett) tests were performed. Data were transformed (square root) if criteria of a normal distribution or variance homogeneity were not met. If a transformation of data did not allow the use of ANOVA, the non-parametric Kruskal-Wallis test was performed. In that case, a multi comparison test from the package pgirmess was used for *post-*hoc comparison. A principal component analysis (PCA) was used to test for the correlations between cellular parameters of *T*. *weissflogii* by using the results from the flow cytometric measurements. To investigate the effect of growth rate on the strain variance, we grouped the observations in equal numbers of observations starting at the low growth rates and plotted the variance of the parameters in these different groups. For this, all negative growth rates were set to zero. We used the libraries *mltools*, *data*.*table*, and *reshape* to group the growth rate for equal numbers of observations and extracted the values of the variance of each group. Analyzing the linear correlation between growth rate and the cell size variability, we used the libraries *lsmeans*, *psych*, and *data*.*table* to test for differences between the slopes and to calculate the Pearson correlation coefficient R^2^ of the six treatments. The significance level of all comparisons was fixed at 95% (p = 0.05).

## Results

### Growth rate and cellular parameters

**Fig 1:**
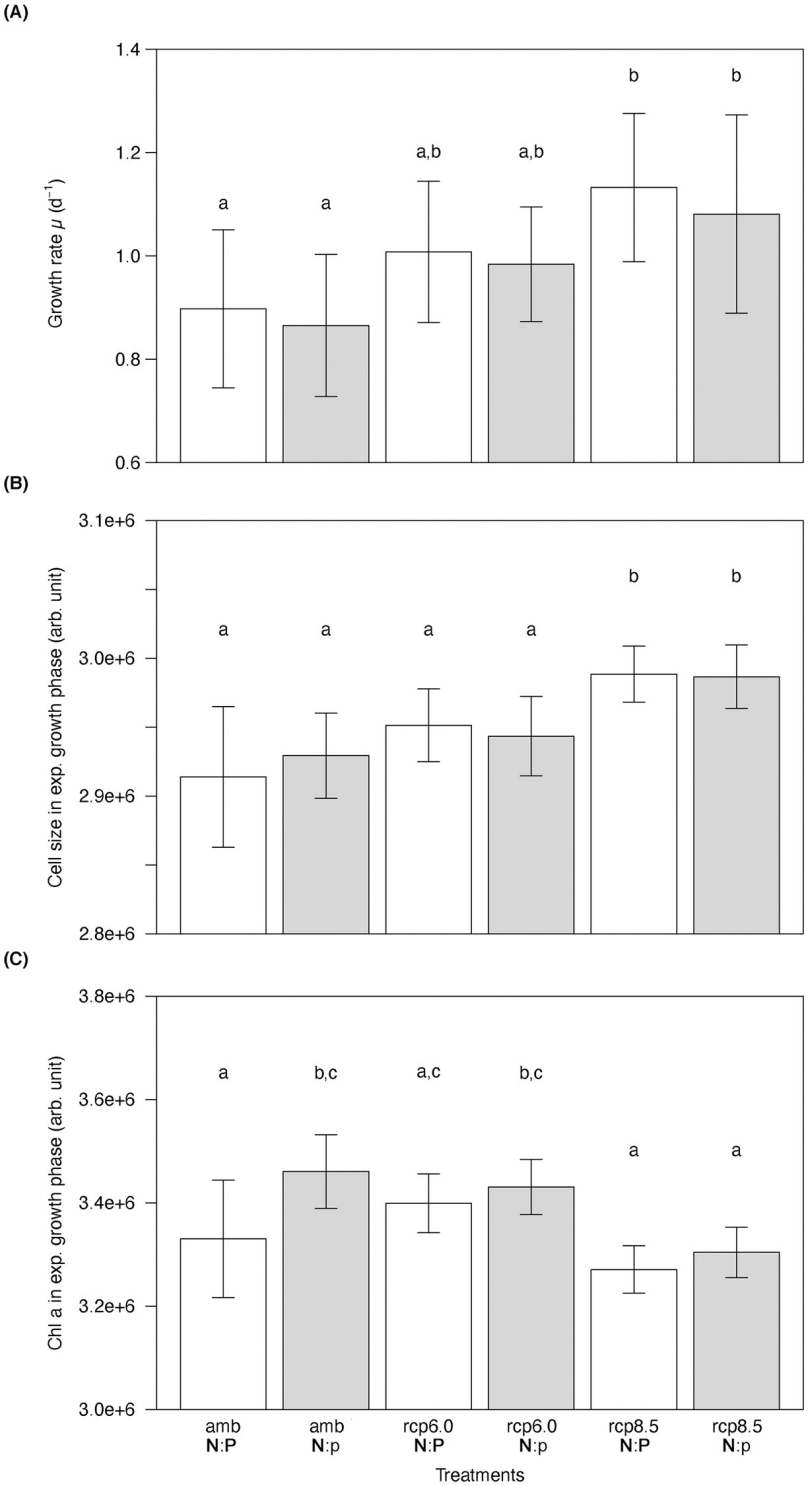
Growth rates (A), cell size (B), and chlorophyll *a* content (C) of *T*. *weissflogii* during the exponential growth phase under three different temperature and pCO_2_ treatments (amb = 14°C, 450ppm; rcp6.0 = 15.5°C, 800ppm; rcp8.5 = 17°C, 1000ppm) crossed with two N:P ratios (N:P = 16; N:p = 25). Data presented are means and standard deviations of six replicates. Different letters (a, b, c) indicate significant differences (two-way ANOVA, p < 0.05). A notation with more than one letter (a,b) means that there is no significant difference with either (a) or (b), but there is a statistically significant difference with (c).

All cultures of *T*. *weissflogii* reached a similar maximum cell density of approximately 51,000 cells ml^-1^ (S1 Fig). The higher temperature and pCO_2_ in the RCP 6.0 and RCP 8.5 scenarios resulted in higher maximum growth rates in the exponential growth phase, and the RCP 6.0 and RCP 8.5 cultures reached the stationary phase one and two days earlier than the ambient cultures, respectively. To identify the effect of the treatments, we plotted the average growth rate during the exponential growth phase. Within the exponential growth phase, the growth rate of *T*. *weissflogii* increased with increasing temperature and pCO_2_ (Fig 1A). Overall, the global change scenarios significantly influenced growth rates during the exponential growth phase (two-way ANOVA, F_2,98_ = 20.99, p < 0.001), with significantly higher growth rates in the RCP 8.5 scenarios than in the ambient scenarios (Tukey’s post-hoc test, p < 0.001). The N:P treatment did not influence growth rates (Tukey’s post-hoc test, p = 0.21). During the exponential growth phase, average cell size was also significantly affected by the global change scenarios (two-way ANOVA, F_2,98_ = 52.59, p < 0.001), with significantly larger cell size in the RCP 8.5 scenarios than in the ambient and the RCP 6.0 scenarios (Tukey’s post-hoc test, p < 0.001), but no effect of the two N:P ratios (Tukey’s post-hoc test, p = 0.91) (Fig 1B). The global change scenarios also affected the average chlorophyll *a* content during the exponential growth phase of the cultures (Fig 1C) (two-way ANOVA, F_2,98_ = 11.69, p = 0.003), with significantly lower cellular chlorophyll *a* content at the RCP 8.5 scenarios and the amb **N**:**P** scenario than in the other scenarios (Tukey’s post-hoc test, p < 0.001).

**Fig 2:**
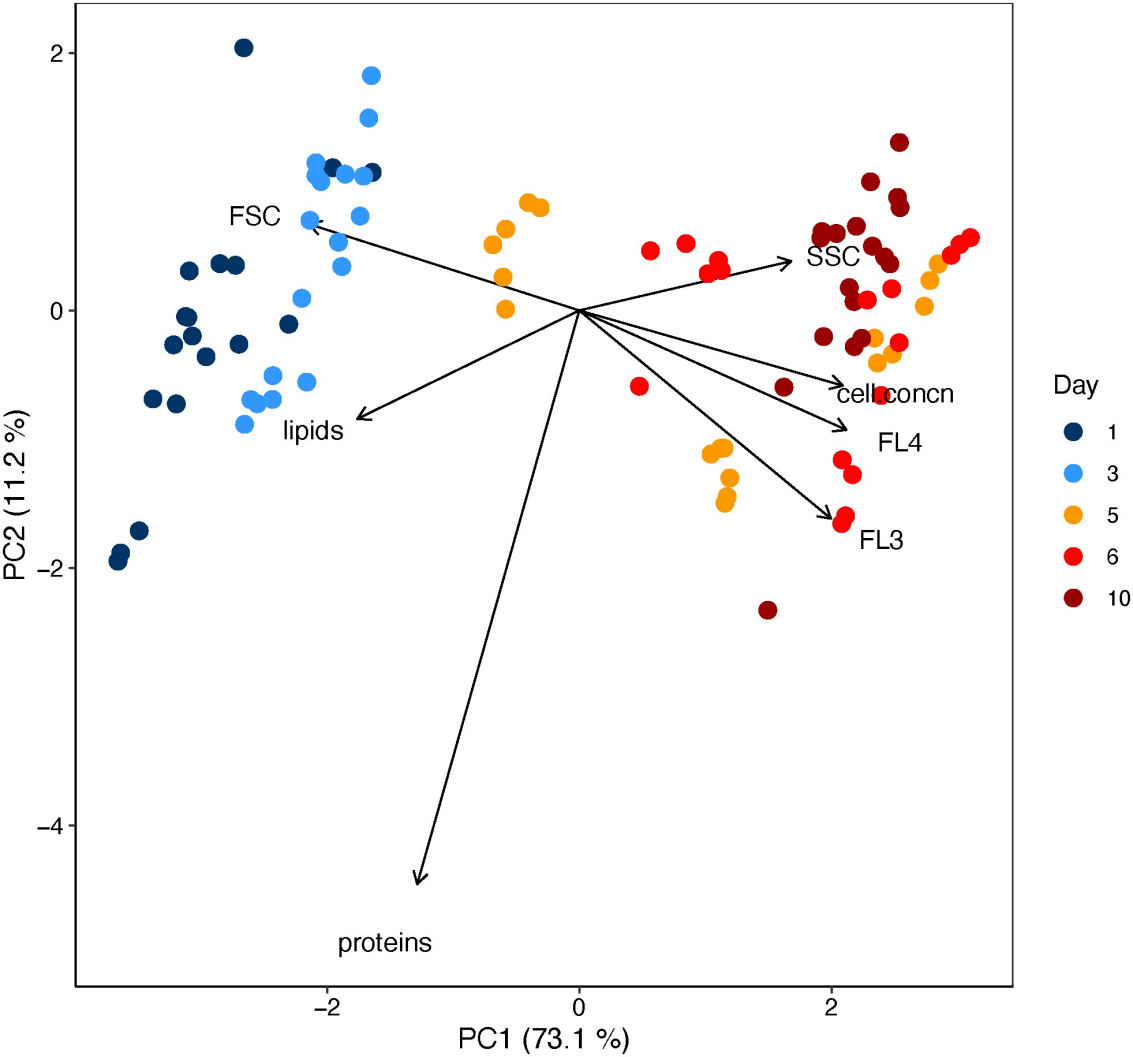
Results of the principal component analysis (PCA) of the two dominant components produced by parameters measured by flow cytometry: Cell concentration, FSC (cell size), SSC (granularity), FL3 and FL4 (chlorophyll *a*), and the staining signal of lipids and proteins. The PCA includes measurements from days 1, 3, 5, 6, 10. The first two days cover the exponential growth phase and the last two the stationary phase of the *T*. *weissflogii* cultures. At day 5 the cultures were in between these two phases. The PCA includes 90 samples.

**Fig 3:**
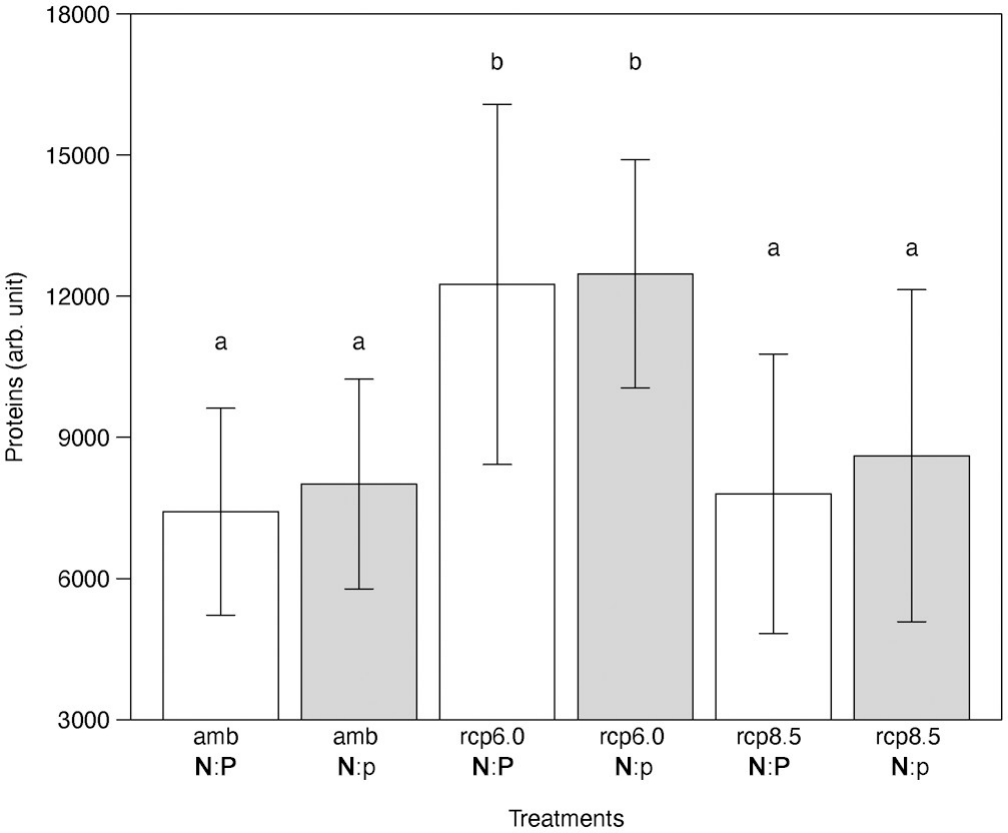
Cellular protein content measured through Fluorescein-5-Maleimide (FITC) staining (detected by FL1) of *T*. *weissflogii* under three different temperature and pCO_2_ treatments (amb = 14°C, 450ppm; rcp6.0 = 15.5°C, 800ppm; rcp8.5 = 17°C, 1000ppm) crossed with two N:P ratios (N:P = 16; N:p = 25). Data presented are means and standard deviations of three replicates. Different letters (a, b) indicate significant differences (two-way ANOVA, p < 0.05).

Using a principal component analysis (PCA), we tested whether the exponential growth and stationary phase of batch cultures can be distinguished by cellular characteristics of *T*. *weissflogii*, and if so, which cellular traits are correlated with the growth phases (Fig 2). The PCA included the mean value of each cellular trait for each sample measured by flow cytometry: cell concentration, forward scatter (FSC, representing cell size), side scatter (SSC, representing the shape and internal complexity or granularity of the cells), as well as signals from the fluorescence detectors (FL3 and FL4) for chlorophyll *a* emission of the diatom cells, and from lipid and protein staining. Along with the first principal component (PC1, 73.1%), two clusters can be identified within the data set. The two clusters characterize the exponential growth phase and the stationary growth phase, respectively, and thus describe growth-related characteristics of *T*. *weissflogii*. The PCA indicates several correlations between cellular traits and growth phases. Cell concentration, SSC, FL3, and FL4 were positive correlated, whereas a negative correlation was detected between these parameters and the FSC as well as cellular lipid content. In contrast, the protein content of the cells did not correlate with growth phases and mainly described PC2 (11.2%). An analysis testing for the effect of the treatments on these parameters, combining exponential growth and stationary phase, showed no or only a marginal significant differences (Kruskal-Wallis _FSC,_ H_5_ = 7.79, p = 0.17; Kruskal-Wallis _FL3_, H_5_ = 11.79, p = 0.037, Kruskal-Wallis _FL4_, H_5_ = 6.60, p = 0.25, Kruskal-Wallis _lipids_, H_5_ = 13.98, p = 0.014). A *post-hoc* comparison identified the marginal significant difference in FL3 between the scenarios amb, **N**:**P** and RCP 6.0, **N**:p, but no significant differences in cellular lipid content between any treatment. Compared with the cellular parameters describing PC1, the differences in cellular protein content were not caused by different growth phases but by the experimental treatments. Indeed, the protein content was significantly higher in both RCP 6.0 **N**:**P** and RCP 6.0 **N**:p scenarios than in the ambient and RCP 8.5 scenarios (Fig 3, two-way ANOVA, F_2,84_ = 21.18, p < 0.001), with no influence of N:P treatments (Tukey’s post-hoc test, p = 0.28).

### Among-strain population and cell-to-cell variability

While the above-described results represent the effect of different abiotic conditions on cellular traits of *T*. *weissflogii*, the following describe the dynamics of cellular traits over different growth phases. We focused on growth rate, including both growth phases of the batch culture, as a master trait of phytoplankton [48], and linked it to the biochemical composition of *T*. *weissflogii* cells by examining changes in size, chlorophyll *a*, lipid, and protein content of the cells.

**Fig 4:**
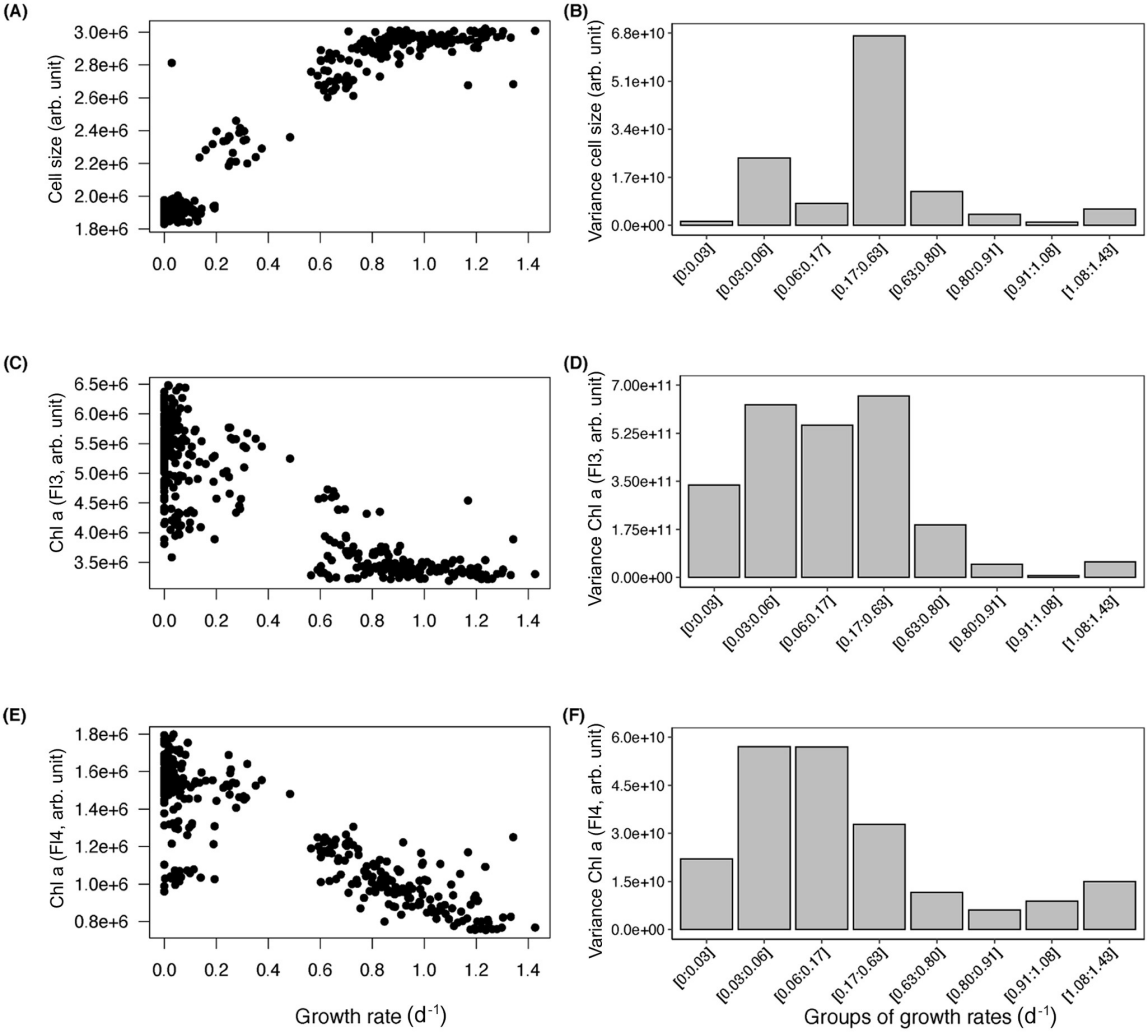
Cell size represented by FSC (A) and chlorophyll *a* content represented by FL3 and FL4 fluorescence (C, E) of *T*. *weissflogii* over growth rates from exponential growth and stationary phase. The variance of these parameters (B, D, F) in growth rates grouped by equal numbers of observations. Data represent the mean values of each triplicate sample measured via flow cytometry at five days.

The response of the FSC, representative of cell size, to increasing growth rates followed a sigmoidal shape with larger cell size at higher growth rates (Fig 4A), whereas the cellular chlorophyll *a* content, as detected by FL3 and FL4, decreased with increasing growth rate (Fig 4C and 4E). Since phytoplankton populations naturally transition quite fast from exponential growth phase to stationary phase, the number of observations was higher at low and high growth rates around 0 and 1 d^-1^ than at intermediate growth rates between 0.2 and 0.6 d^-1^. Therefore, we created bins of an equal number of observations to test for changes in variability as a function of growth rates. While the among-strain population variability, i.e., the variation between cultures, of the FSC signal did not vary with growth rate (Fig 4B), the variability in cellular chlorophyll *a* content decreased with increasing growth rate (Fig 4D and 4E). The variance of cellular protein contents, detected by FITC stain, showed no clear trend over a range of growth rates (S2B and S2D Fig). The variance of cellular lipid content, in contrast to the other measured parameters, was higher in the exponential growth phase than in the stationary phase (S2C Fig).

**Fig 5:**
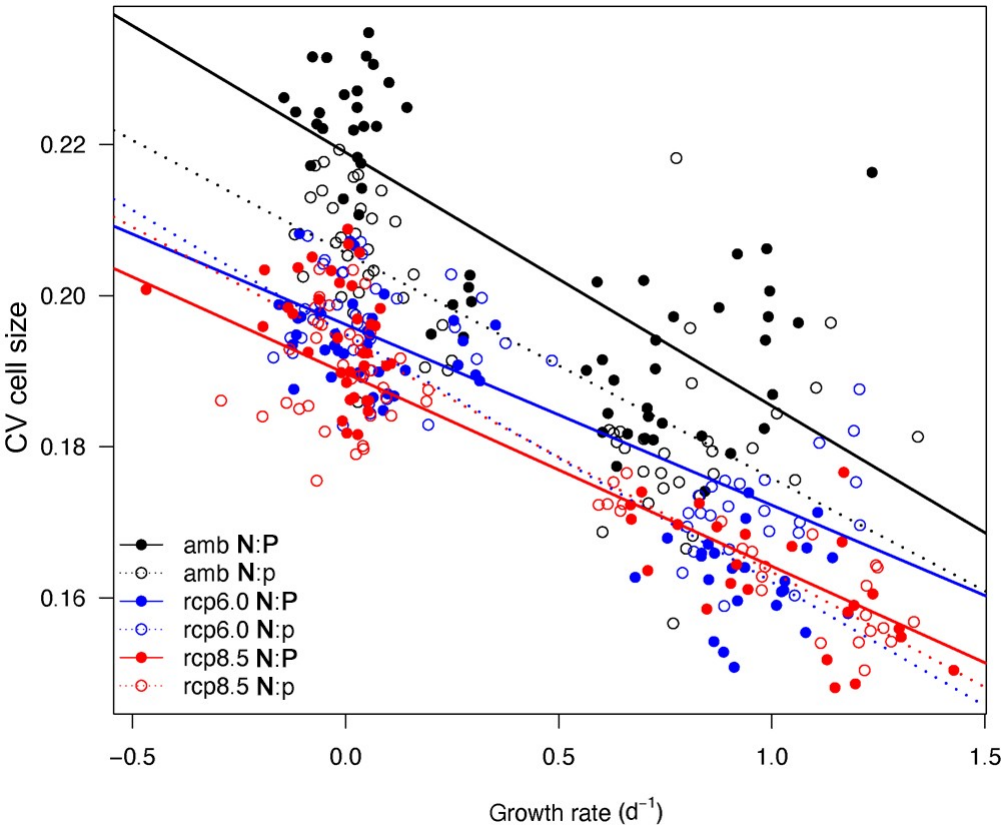
Linear regression model of cell size variability (CV FSC) over growth rate of *T*. *weissflogii* from exponential growth and stationary phase; data represented for each treatment are mean values of six replicates. The R^2^ (Pearson correlation coefficient), representing the fit of the model, indicated an increase in linear correlation between cell size variability and growth rate with increasing treatment intensity: From ambient (R^2^_amb;N:P_ = 0.76, R^2^_amb;N:p_ = 0.76) to RCP 6.0 (R^2^_rcp6.0;N:P_ = 0.83, R^2^_rcp6.0;N:p_ = 0.92) and to RCP 8.5 (R^2^_rcp8.5;N:P_ = 0.91, R^2^_rcp8.5;N:p_ = 0.92). Regression lines of the ambient treatments are significantly different from the RCP 6.0 and RCP 8.5 treatments (p ≤ 0.029).

To assess the impact of concurrent changes in abiotic parameters on the phenotypic plasticity of *T*. *weissflogii*, we further tested for a correlation between cell-to-cell variability within strain populations, i.e., differences between single cells within a culture as captured by the coefficient of variation (CV), and growth rate. Differences in cell-to-cell variability were observed only for cell size (FSC). Growth rate and CV of FSC were negatively correlated and followed a linear model (Fig 5). When comparing the regression lines of the six treatments with a pairwise comparison test, we observed an increase in the strength of the correlation between growth rate and CV of FSC with treatment intensity, indicated by an increase in Pearson correlation coefficient R^2^ from 0.76 in both ambient scenarios to 0.92 in the future scenarios RCP 6.0 and RCP 8.5 with **N**:p = 25 (Fig 5). Additionally, we observed a significant interaction in the relationship between growth rate and CV of FSC (two-way ANOVA, F_5,348_ = 9.27, p < 0.001), and a pairwise comparison of the slopes identified significantly higher cell size cell-to-cell variability within single strain populations in the ambient scenarios than in the global change scenarios RCP 6.0 and RCP 8.5 (p ≤ 0.029).

## Discussion

### Growth rate and cellular parameters

Future increases in surface water temperature and pCO_2_ may lead to increased metabolic activity and growth of phytoplankton [reviewed by 25]. The results of our study are in line with these predictions, and we observed that an increase in temperature combined with higher pCO_2_ can enhance the growth of *T*. *weissflogii*. Testing realistic scenarios with temperature increases of +1.5 and +3°C enabled us to identify that phytoplankton respond to even relatively small changes in the environment. Moreover, an association between growth and other functional traits can often be found [9]. These phytoplankton traits may be affected not only directly by changes in temperature and pCO_2_, but also indirectly by increasing growth rates. The absence of significant differences between the two N:P ratios may be due to a relatively short incubation time, as the batch culture approach used in this study did not leave much time for the phytoplankton cells to acclimate to culture conditions. Additionally, the absence of differences between the N:P scenarios may indicate that the supply of N and P may have been sufficient for growth in both treatments. However, our results show that growth rate is a master trait, which is significantly correlated with multiple other traits. For instance, we identified a positive correlation between growth rate and cell size of *T*. *weissflogii*. In the exponential growth phase, both growth rate and cell size increased with increasing temperature and pCO_2_. As the diatom populations grew slower during the transition from exponential growth to stationary phase, cells became smaller, too. The correlation between cell size and growth rate has been investigated before and seems to be species dependent [35,49]. Additionally, during asexual reproduction, cell division produces progressively smaller diatom cells until a certain threshold at which sexual reproduction takes place, allowing cells to regain a larger size. Amato et al. [50] as well as Chisholm and Costello [51] reported that cell size in *Coscinodiscus pavillardii* and *Pseudo-nitzschia delicatissima* decreases significantly with increasing growth rate. Our results are also supported by other studies on *T*. *weissflogii*, which observed a strong positive relationship between growth rate and cell size when different subclones of the same isolate were compared [33,51]. To calculate the percentage of decrease in cell size, we used the FSC signal from the flow cytometer and observed a 33–36% decrease in size from exponential growth to stationary phase. According to Olenina et al. [52], *T*. *weissflogii* can be found in three size classes (12–15 μm, 15–18 μm, 18–22 μm), covering the same range of variation as the one we recorded. Thus, the relationship between growth rate and cell size appears to be species-specific and has been shown to be a function of the balance between sexual and asexual reproduction [33]. Moreover, smaller cells have a higher surface area to volume ratios, which enhances nutrient transport capacity under low dissolved nutrient conditions [53,54]. Therefore, it is possible that the decrease in cell size from exponential growth to stationary phase we recorded is a strategy by *T*. *weissflogii* to increase its nutrient uptake efficiency and competitive ability under decreasing nutrient concentrations.

We also observed a negative correlation between growth rate and cellular chlorophyll *a* content. At higher growth rates, the chlorophyll *a* content of *T*. *weissflogii* decreased slightly in the RCP 8.5 scenario. This result contradicts phytoplankton ecophysiology studies conducted in chemostats that report generally higher chlorophyll *a* content in faster-growing phytoplankton populations [55,56]. However, in our experiment, we used batch cultures and the populations transitioned from exponential growth to stationary phase. Cell densities increased while growth rates decreased. At high cell densities, phytoplankton cells can increase their chlorophyll *a* content to compensate for self-shading [57]. Therefore, cellular chlorophyll *a* content should be related not only to growth rate but also to cell density.

Interestingly, the cellular protein content of *T*. *weissflogii* was not correlated with its growth rate. Instead of an increase or decrease of cellular protein content with increasing growth rate, we recorded an overall higher cellular protein content in the RCP 6.0 scenario than in the ambient, but not in the RCP 8.5 scenario. Toseland et al. [58] found a similar dependence of cellular protein content to temperature, with increased protein synthesis as temperature increased, and suggested the temperature dependence of ribosomal translation as a likely explanation. However, it is unclear why the cellular protein content of *T*. *weissflogii* was higher in the RCP 6.0 scenario than in the RCP 8.5 scenario. The lipid content of T. *weissflogii* was higher at the beginning of the experiment and decreased from the exponential growth phase to the stationary phase. Other studies have reported relatively low lipid content under optimal growth conditions [reviewed by 59] or an accumulation of lipids when diatom cultures are nutrient limited, especially under N-limitation [60,61]. As mentioned above, the absence of differences between the N:P scenarios may indicate that the supply of N and P may have been sufficient for growth, which explains the patterns of cellular lipid content of *T*. *weissflogii* we observed here.

### Among-strain populations and cell-to-cell variability

In a meta-analysis conducted with 55 data sets, Hillebrand et al. [18] assessed the correlation between stoichiometry and growth rate of several phytoplankton species and observed that higher growth rates constrained the N:P stoichiometry of phytoplankton cells. Here, we hypothesized that among-strain population variability in functional traits, which describes the variation within one strain growing at different environmental conditions, as well as cell-to-cell variability within a single strain population of phytoplankton, representing the phenotypic variability of one culture, decrease with increasing growth rate, not only between species [18] but also within one strain. Our results partially support this hypothesis. While the variability in cellular protein content was not affected by growth rate, neither on the single strain population level nor on the cell-to-cell level, the among-strain population variability in cellular lipid content was higher at higher growth rates. However, the higher variability was most likely caused by the overall higher cellular lipid content at high growth rates compared with the lipid content at low growth rates. In contrast, the among-strain population variability in cellular chlorophyll *a* content decreased significantly with increasing growth rates, and the cell-to-cell variability in cell size also decreased with increasing growth rate. Whereas the among-strain population variability indicates that faster growth channels the chlorophyll *a* content to an optimal budget, as stated by Hillebrand et al. [18], growth rate did not influence the chlorophyll *a* cell-to-cell variability within a single strain population, which was constant overall. A possible explanation for this could be that not only in our experiment but also in nature phytoplankton cells experience slightly different light conditions, since light is supplied from an angle that does not allow homogenous irradiation, causing some degree of phenotypic plasticity in cellular chlorophyll *a*. On the other hand, the constant strain variability in cell size suggests that cell size is linked only to growth rate and not to environmental parameters, supporting the idea that changes in cell size with growth rates are caused by the reproduction of diatom cells. We also observed a decrease in cell-to-cell variability in cell size with increasing growth rates, i.e., at higher growth rate, cells within a single strain culture become more similar in cell size. Additionally, the global change scenarios RCP 6.0 and 8.5 resulted in an overall lower cell-to-cell variability in cell size, which did not only hold true during the exponential growth phase but was also maintained during the stationary phase. Hence, it seems that the degree of variability in cell size within a single strain population develops at fast growth and is maintained as the growth rate decreases. This second type of variability, the cell-to-cell variability, can only be explained through phenotypic plasticity. High phenotypic plasticity in cell size can be advantageous to cope with shifts in environmental conditions, whether abiotic parameters or predation pressure, and it is indeed a key aspect determining the ability of phytoplankton to respond to environmental changes [reviewed by 6]. Since it is directly correlated to nutrient uptake efficiency and resistance to grazers [53], cell size is an important trait of phytoplankton. However, phenotypic plasticity responses have received little focus from global change studies, and, as far as we know, our study is the first to investigate not only the variability among single strain populations but also the cell-to-cell variability of phytoplankton functional traits in a global change context.

## Conclusion

In this study, we observed that realistic future global change scenarios significantly influence the growth rate of the diatom species *T*. *weissflogii*. We also identified strong correlations between growth rate and cellular traits. Growth rate was positively correlated with cell size and cellular lipid content, and negatively correlated with cellular chlorophyll *a* content. We have shown that trait variability among single strain populations as well as cell-to-cell variability within a single strain population decrease as growth rate increases. Independent of growth rate, higher temperature and pCO_2_ also caused an overall decrease in cell size cell-to-cell variability. Future studies should therefore focus on the interaction between abiotic conditions, growth rate, and phenotypic trait plasticity of other phytoplankton species to evaluate the impact of global change.

## Supporting information

S1 Fig Growth curve of *T*. *weissflogii* under three different temperature and pCO_2_ treatments: ambient = 14°C, 450ppm (black); RCP 6.0 = 15.5°C, 800ppm (blue); RCP 8.5 = 17°C, 1000ppm (red) crossed with two N:P ratios: N:P = 16 (filled circles); N:p = 25(open circles). Data presented are means and standard deviations of six replicates.
(TIF)

S2 Fig Lipid content, measured through NILE RED staining (detected by FL2 and FL3), (A) and protein content, measured through Fluorescein-5-Maleimide (FITC) staining (detected by FL1), (C) of *T*. *weissflogii* over growth rate, and variance of these parameters (B, D) in growth rates grouped by equal numbers of observations. Data represent the mean values of each triplicate sample measured via flow cytometry at five days.
(TIF)

S1 Table Dissolved inorganic nitrogen (DIN) and phosphorus (DIP) concentration in μmol L^-1^ sampled at day 5 (early stationary phase) and day 10 (late stationary phase) of the batch culture experiment. (DOCX)
