# Supplementary figures and images for: Environmental impacts on single-cell variation within a ubiquitous diatom: The role of growth rate
Source: PLoS One. 2021 May 7;16(5):e0251213. doi: 10.1371/journal.pone.0251213 (PMC8104383; doi:10.1371/journal.pone.0251213)

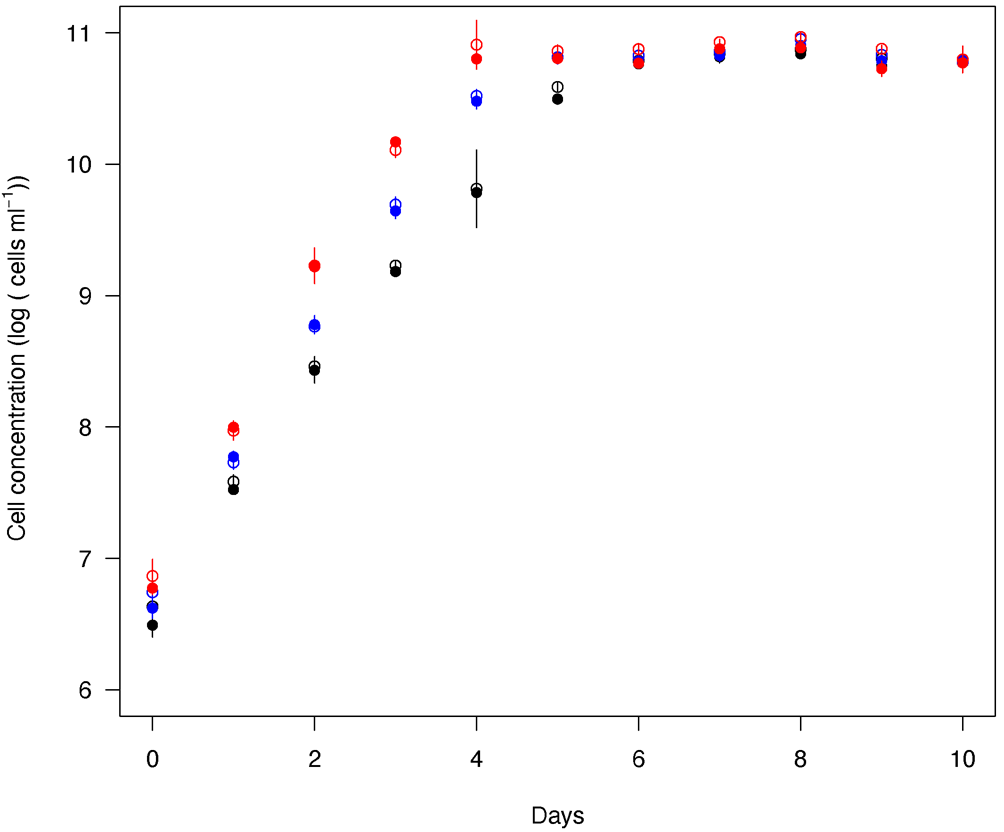

Supplement: S1 Fig — Data presented are means and standard deviations of six replicates. (TIF) [file pone.0251213.s001.tif]

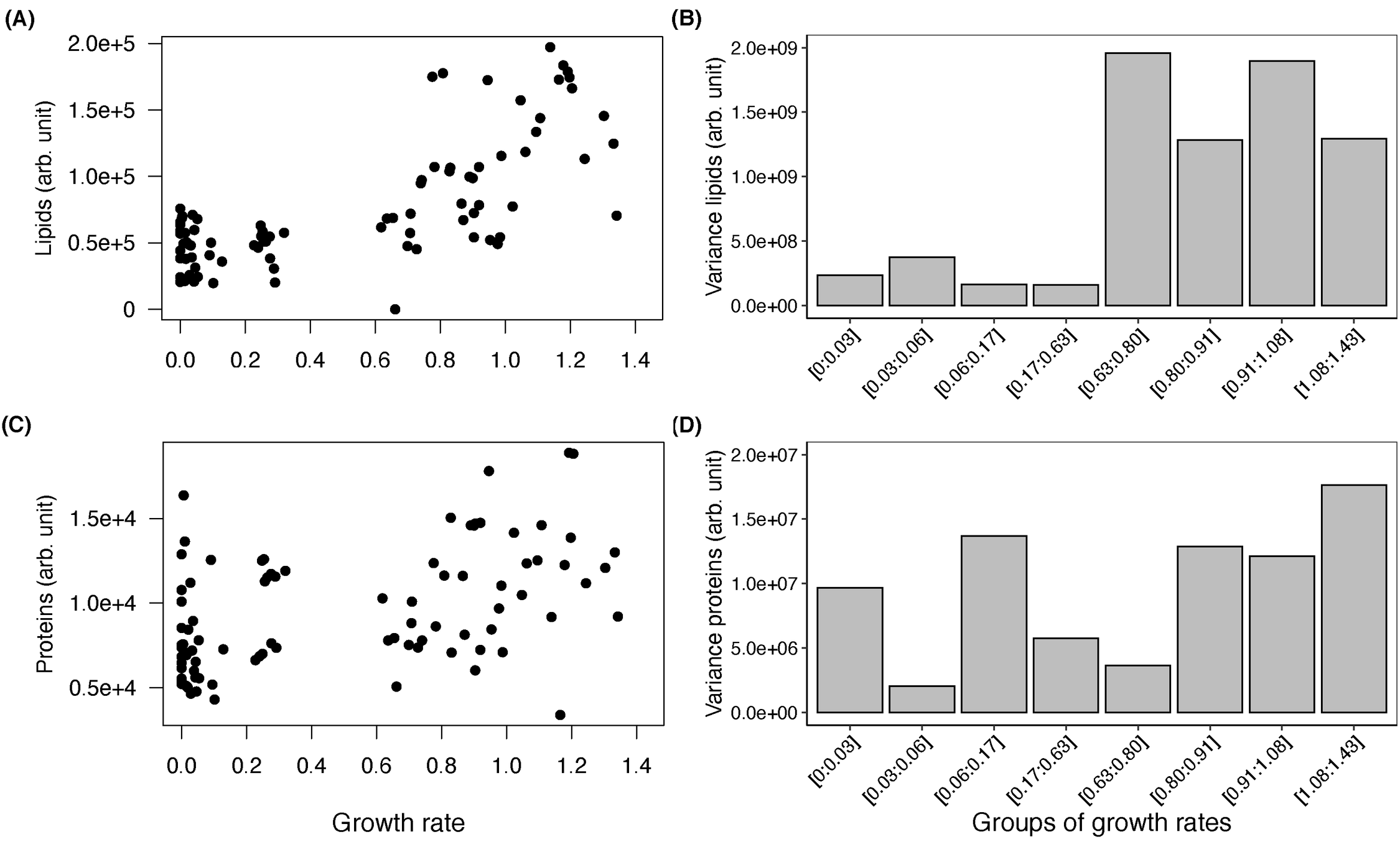

Supplement: S2 Fig — Data represent the mean values of each triplicate sample measured via flow cytometry at five days. (TIF) [file pone.0251213.s002.tif]
