# Supplementary material for: Environmental impacts on single-cell variation within a ubiquitous diatom: The role of growth rate
Source: PLoS One. 2021 May 7;16(5):e0251213. doi: 10.1371/journal.pone.0251213 (PMC8104383; doi:10.1371/journal.pone.0251213)
Supplement: S1 Table — (DOCX) [file pone.0251213.s003.docx]

S1 Table: Dissolved inorganic nitrogen (DIN) and phosphorus (DIP) concentration in µmol L^-1^ sampled at day 5 (early stationary phase) and day 10 (late stationary phase) of the batch culture experiment.

| DIN (µmol L^-1^) | amb N:P | amb N:p | rcp6.0 N:P | rcp6.0 N:p | rcp8.5 N:P | rcp8.5 N:p |
| --- | --- | --- | --- | --- | --- | --- |
| Day 5 | 379.10 | 338.45 | 402.30 | 418.40 | 388.05 | 302.90 |
| Day 10 | 250.80 | 270.80 | 285.05 | 226.65 | 386.95 | 312.55 |
|  |  |  |  |  |  |  |
| DIP (µmol/L) | amb N:P | amb N:p | rcp6.0 N:P | rcp6.0 N:p | rcp8.5 N:P | rcp8.5 N:p |
| Day 5 | 29.30 | 14.95 | 27.65 | 16.20 | 29.65 | 17.25 |
| Day 10 | 21.20 | 16.45 | 17.95 | 15.70 | 11.95 | 12.65 |
